# The link between orthology relations and gene trees: a correction perspective

**DOI:** 10.1186/s13015-016-0067-7

**Published:** 2016-04-16

**Authors:** Manuel Lafond, Riccardo Dondi, Nadia El-Mabrouk

**Affiliations:** Département d’informatique et de recherche opérationnelle, Université de Montréal, Montreal, QC Canada; Dipartimento di Scienze Umane e Sociali, Università degli Studi di Bergamo, Via Donizetti 3, 24129 Bergamo, Italy

**Keywords:** Orthology, Paralogy, NP-Hardness, Gene tree, Species tree

## Abstract

**Background:**

While tree-oriented methods for inferring orthology and paralogy relations between genes are based on reconciling a gene tree with a species tree, many tree-free methods are also available (usually based on sequence similarity). Recently, the link between orthology relations and gene trees has been formally considered from the perspective of reconstructing phylogenies from orthology relations. In this paper, we consider this link from a correction point of view. Indeed, a gene tree induces a set of relations, but the converse is not always true: a set of relations is not necessarily in agreement with any gene tree. A natural question is thus how to minimally correct an infeasible set of relations. Another natural question, given a gene tree and a set of relations, is how to minimally correct a gene tree so that the resulting gene tree fits the set of relations.

**Results:**

We consider four variants of relation and gene tree correction problems, and provide hardness results for all of them. More specifically, we show that it is NP-Hard to edit a minimum of set of relations to make them consistent with a given species tree. We also show that the problem of finding a maximum subset of genes that share consistent relations is hard to approximate. We then demonstrate that editing a gene tree to satisfy a given set of relations in a minimum way is NP-Hard, where “minimum” refers either to the number of modified relations depicted by the gene tree or the number of clades that are lost. We also discuss some of the algorithmic perspectives given these hardness results.

## Background

Genes, the molecular units of heredity, hold the information to build and maintain cells. In the course of evolution, they are duplicated, lost, and passed to organisms through speciation. Genes originating from the same ancestral copy are called *homologs*. Homologous gene are grouped into *gene families*, usually via sequence similarity methods. Moreover, homologous genes can be *orthologous*, if their parental origin is a speciation, or *paralogous*, if it is a duplication. Orthologous gene are considered to be more similar in function than paralogs, a conjecture known as the *orthology conjecture* [1]. This is a major motivation for inferring gene evolution, as it is a prerequisite for functional prediction purposes.

Starting usually from a DNA or protein sequence alignment, the tree-based method requires to build a phylogenetic tree, called gene tree, for the considered gene family. Reconciliation [2] with the species tree then allows to infer evolutionary events (duplications and speciations) associated with the internal nodes of the gene tree. Hence the internal nodes of a gene tree can be labeled as duplications and losses, and such a labeling induces a full orthology and paralogy set of relations between gene pairs. In order to detect orthology, tree-free methods are also available. These methods are based on gene clustering according to sequence similarity, (cf. e.g. the COG database [3], OrthoMCL [4], InParanoid [5], Proteinortho [6]), synteny [7, 8] or functional annotation of genes [9]. Such methods usually are not able to detect a full set of relations, but only a partial set, i.e. some relations among genes are not inferred.

Recent papers [10, 11] have investigated, from a graph theory point of view, the link between trees and orthology/paralogy relations (we just say “relations” in the following). Given a gene family $\Gamma$ and a set $\mathcal{C}$ of pairwise relations, a first problem is whether we can reconstruct a labeled gene tree for $\Gamma$ inducing $\mathcal{C}$. The problem can be subdivided into two parts. First, we can consider whether $\mathcal{C}$ is *satisfiable*, i.e. whether there exists an event-labeled gene tree *G* in agreement with $\mathcal{C}$. However satisfiability is not sufficient to ensure the possibility for the relation set to reflect a true history, as nodes of *G* labeled as speciations can be contradictory. This raises the second question which is the existence of an *S-consistent* gene tree, namely an event-labeled tree that can be obtained by reconciliation with a species tree *S*. A simple characterization of satisfiability is given in [10], when the set $\mathcal{C}$ is a full set of relations (i.e. each pair of genes of $\Gamma$ is in ${\mathcal C}$). On the other hand, checking for *S*-consistency can be done in polynomial-time for full sets [12, 13], and also partial sets of relations [14].

In this paper we explore the link between relations and trees in the perspective of relation and tree correction. Several gene tree databases from whole genomes are available, including for instance Ensembl Compara [15], Hogenom [16], Phog [17], MetaPHOrs [18], PhylomeDB [19], Panther [20]. However, due to various limitations such as alignment errors, systematic artifacts of inference methods or insufficient differentiation between sequences, trees are known to contain errors and uncertainties. Consequently, a great deal of effort has been put towards tools for gene tree editing [21–29]. Most of them are based on selecting, in a neighborhood of an input tree, one best fitting the species tree.

Two years ago, we developed the first algorithm for gene tree correction using orthology relations [7]. Here we address, from a complexity and approximation point of view, the more general problem of correcting a gene tree according to a set of orthology and paralogy relations. We consider two objective functions: the number of unchanged relations (from orthology to paralogy or vice-versa), leading to the Maximum Homology Correction problem, and the number of unchanged clades (the Robinson-Foulds distance [30]), leading to the Maximum Clade Correction problem. We provide NP-completeness results for these two problems.

Conversely, we also address the problem of correcting a set of relations so that it represents a valid history in terms of *S*-consistency. A set of relations is usually represented as a graph *R*, where edges represent orthologous relations and non-edges represent paralogous relations. The satisfiability problem related to *S*-consistency reduces to adding or removing a minimum number of edges of *R* in order to make it $P_4$-free (that is, it contains no induced path of length three), as shown in [10]. The problem is known to be NP-Hard and fixed parameter tractable [31]. In [11], an integer linear programming formulation is used to correct relation graphs of reasonable size. A factor approximation algorithm of factor $4 \Delta$, where $\Delta$ is the degree of the graph *R*, is given in [32]. The *S*-consistency problem, however, has never been studied.

In this paper, two criteria are considered for correcting a set *R* of relations: minimize the number of modified relations, and maximize the number of genes inducing an *S*-consistent set of relations. The first problem is shown to be NP-complete, while the second problem is shown to be not approximable within factor $d n^{\frac{1}{2}(1-\varepsilon )},$ for any $0 < \varepsilon < 1$ and any constant $d > 0$.

## Trees and orthology relations

All trees considered in this paper are assumed to be rooted. They are not necessarily binary, but we assume that all nodes are of degree at least three, except possibly the root that can be of degree two. Given a set *X*, a *tree T for X* is a tree whose leafset ${\mathcal L}(T)$ is in bijection with *X*. We denote by *V*(*T*) the set of nodes and by *r*(*T*) the root of *T*. Given an internal node *u* of *T*, the subtree rooted at *u* is denoted $T_u$ and we call the leafset ${\mathcal L}(T_u)$ the *clade ofu*. A node *u* is an *ancestor* of *v* if *u* is on the (inclusive) path between *v* and the root, and we then call *v* a descendant of *u*. If $u \ne v$, then *v* is a *strict descendant* of *u*, and if *u* and *v* are connected by an edge of *T*, then *v* is a *child* of *u*. The *lowest common ancestor* (lca) of *u* and *v*, denoted $lca_T(u, v)$, is the ancestor common to both nodes that is the most distant from the root. We say that *u* and *v* are *separated* if and only if $lca_T(u, v) \notin \{u,v\}$ (i.e. none is an ancestor of the other). We define $lca_T(U)$ analogously for a set *U* of nodes. Let $L'$ be a subset of ${\mathcal L}(T)$. The restriction $T|_{L'}$ of *T* to $L'$ is the tree with leaf set $L'$ obtained from the subtree of *T* rooted as $lca_T(L')$, by removing all leaves that are not in $L'$, and contracting all internal nodes of degree two, except the root. Let $T'$ be a tree such that ${\mathcal L}(T') = L' \subseteq {\mathcal L}(T)$. We say that *T* displays $T'$ if and only if $T|_{L'}$ is $T'$.

### Evolution of a gene family

Species evolve through *speciation*, which is the separation of one species into distinct ones. A species tree *S* for a species set $\Sigma$ represents an ordered set of speciation events that have led to $\Sigma$: an internal node is an ancestral species at the moment of a speciation event, and its children are the new descendant species. Inside the species’ genomes, genes undergo speciation when the species to which they belong do, but also duplications, and losses (other events such as transfers can happen, but we ignore them here). A *gene family* is a set of genes $\Gamma$ accompanied by a *mapping function*$s : \Gamma \rightarrow \Sigma$ mapping each gene to its corresponding species. The evolutionary history of $\Gamma$ can be represented as a node-labeled *gene tree* for $\Gamma$, where each internal node refers to an ancestral gene at the moment of an event (either speciation or duplication), and is labeled as a speciation (*Spec*) or duplication (*Dup*) accordingly.

**Fig. 1:**
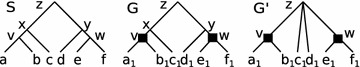
A species tree *S*, a binary *DS*-tree *G* and a non-binary *DS*-tree $G'$. In *DS*-trees, *Dup* nodes are indicated by* squares*. All other nodes are speciations nodes. Each leaf $\alpha _i$ denotes a gene belonging to the genome $\alpha$. *G* is a refinement of $G'$ such that ${\mathcal O} (G) = {\mathcal O} (G')$ and ${\mathcal P} (G) = {\mathcal P} (G')$. Notice that, although in this example the gene trees contain exactly one gene copy from each genome, this is not a requirement. Another example with multiple gene copies in genome *a* is given in Fig. 2

Formally, we call a *DS-tree* for $\Gamma$ a pair $(G, ev_G)$, where *G* is a tree with ${\mathcal L}(G) = \Gamma$, and $ev_G:V(G) \setminus {\mathcal L}(G) \rightarrow \{Dup, Spec\}$ is a function labeling each internal node of *G* as a duplication or a speciation node (we drop the *G* subscript from $ev_G$ when it is clear from the context). Given a species tree *S*, the *LCA-mapping* function $s_G : V(G) \rightarrow V(S)$ maps each gene of *G*, ancestral or extant, to a species as follows: if $g \in {\mathcal L}(G)$, then $s_G(g) = s(g)$; otherwise, $s_G(g) = lca_S( \{s(g') : g' \in {\mathcal L}(G_g) \})$. An example is given in Fig. 1, where the label of each node of *G* represents its LCA-mapping with respect to *S*.

According to the Fitch [33] terminology, we say that two genes *x*, *y* of $\Gamma$ are *orthologous inG* if $ev(lca_G(x, y)) = Spec$, and *paralogous inG* if $ev(lca_G(x, y)) = Dup$. We denote by ${\mathcal O} (G)$, respectively ${\mathcal P} (G)$, the set of all gene pairs that are orthologous, respectively paralogous in *G*. By $xy \in {\mathcal O} (G)$ we mean $\{x, y\} \in {\mathcal O} (G)$ (the same applies for ${\mathcal P} (G)$). In Fig. 1, $a_1c_1 \in {\mathcal O} (G)$ while $a_1b_1 \in {\mathcal P} (G)$. We say that $a_1c_1$ (respec. $a_1b_1$) is an orthology (respec. paralogy) relation *induced* by *G*.

While a history for $\Gamma$ can be represented as a *DS*-tree, the converse is not always true, as a *DS*-tree *G* for $\Gamma$ does not necessarily represent a valid history. For this to hold, any speciation node of *G* should reflect a clustering of species in agreement with *S* [14]. Formally *G* should be *S-consistent*, as defined below.

#### **Definition 1**

Let *S* be a species tree and *G* be a DS-tree. Let *v* be an internal node of *G* such that $ev(v) = Spec$. Then the speciation node *v* is *S-consistent* if and only if for any two distinct children $v_1, v_2$ of *v*, $s_G(v_1)$ and $s_G(v_2)$ are separated in *S*.

We say that *G* is *S-consistent* if and only if every speciation node of *G* is *S*-consistent.

Notice that *G* and *S* are not required to be binary. In particular, the definition of *S*-consistency for a speciation node *v* of *G* does not require *v* to be binary, even if *S* is binary. The reason is that in such a case, one can “refine” *v* into a set of binary *S*-consistent speciation nodes based on the topology of *S*. This operation does not affect the orthology and paralogy relations of the genes of *G* (see Fig. 1). Duplication nodes can be refined as well. Lemma 1 formalizes this intuition. This will serve to show that our results hold for both non-binary and binary gene trees.

#### **Lemma 1**

*Let G be an S-consistent DS-tree for some binary species tree S. Then there is a binary DS-tree*$G'$* such that *$G'$*is S-consistent, and such that*${\mathcal O} (G) = {\mathcal O} (G')$* and*${\mathcal P} (G) = {\mathcal P} (G')$.

#### Proof

Let *v* be a highest non-binary node (i.e. *v* has no non-binary ancestors) of *G* with children $v_1, \ldots , v_k$. We show that *v* can be made to be binary while preserving ${\mathcal O} (G)$ and ${\mathcal P} (G)$, which suffices to prove the Lemma since we can repeat this operation successively on every non-binary node.

If $ev_G(v) = Dup$, obtain a *DS*-tree $G^*$ by removing $v_2, \ldots , v_k$ from the children of *v*, adding a child $v'$ to *v* and adding $v_2, \ldots , v_k$ as children of $v'$, setting $ev_{G^*}(v') = Dup$. Notice that $s_G(w) = s_{G^*}(w)$ for every $w \in V(G) \cap V(G^*) = V(G^*) \setminus \{v'\}$, implying that all speciations remain *S*-consistent. It is readily seen that ${\mathcal O} (G) = {\mathcal O} (G^*)$ and ${\mathcal P} (G) = {\mathcal P} (G^*)$.

If instead $ev_G(v) = Spec$, let $s_1, s_2 \in V(S)$ be the two children of $s_G(v)$. Let $V_j = \{v_i : s_j$ is an ancestor of $s_G(v_i)$, $1 \le i \le k\}$ for $j \in \{1, 2\}$. Notice that for any child $v_i$ of *v*, $s_G(v_i)$ is a strict descendant of $s_G(v)$. For if not, *v* has a child $v_i$ such that $s_G(v_i) = s_G(v)$. But since *v* is a speciation, $s_G(v_i) = s_G(v)$ is separated from $s_G(v_j)$ for any $j \ne i$, implying that $s_G(v)$ is not the lca of $s_G(v_i)$ and $s_G(v_j)$, contradicting the definition of $s_G$. This strict descendant condition implies that $\{V_1, V_2\}$ partitions $\{v_1, \ldots , v_k\}$. Also observe that $V_1$ and V_2 cannot be empty, for otherwise $s_G(v)$ would be equal to either $s_1$ or $s_2$. Obtain $G^*$ by removing the children of *v*, adding two children $w_1$ and $w_2$ to *v*, then adding $V_1$ as children of $w_1$ and $V_2$ as children of $w_2$. Set $ev_{G^*}(v) = ev_{G^*}(w_1) = ev_{G^*}(w_2) = Spec$. Note that the children of $w_1$ and $w_2$ are still from separated species, and so both are *S*-consistent. As for *v*, by the definition of $V_1$ and $V_2$, $s_{G^*}(w_1)$ is a descendant of $s_1$ and $s_{G^*}(w_2)$ is a descendant of $s_2$ (not necessarily a strict descendant). Therefore, both are separated and so *v* is *S*-consistent. The species for every other node remaining unchanged, we conclude that $G^*$ preserves *S*-consistency and does not modify ${\mathcal O} (G)$ nor ${\mathcal P} (G)$. $\square$

We can verify that both *DS*-trees in Fig. 1 are *S*-consistent. For example, the speciation node *z* in $G'$ has children from species *v*, *c*, *d* and *w*, which are pairwise separated in *S*. Notice that, from Definition 1, if *G* is a *DS*-tree, then the lca of two leaves of *G* belonging to the same species must be a duplication node. The converse is not true. For example, in the *S*-consistent gene tree *G* of Fig. 1, the parental node of $e_1$ and $f_1$ is a duplication node even though $e_1$ and $f_1$ belong to two different species.

### Relation graph

A set of orthology/paralogy relations on $\Gamma$ (or simply a relation set) is a pair $C = (C_O, C_P)$ of subsets $C_O, C_P \subseteq {\Gamma \atopwithdelims ()2}$ such that $C_O \cap C_P = \emptyset$ and if $s(x) = s(y)$, then $\{x, y\} \in C_P$. The relation set is said *full* if $C_O \cup C_P = {\Gamma \atopwithdelims ()2}$. A *DS*-tree *G* induces a full set $({\mathcal O} (G), {\mathcal P} (G))$ of relations.

**Fig. 2:**
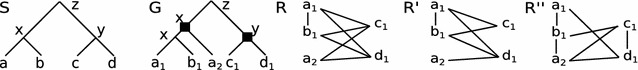
A species tree *S* and a *DS*-tree *G* which is *S*-consistent. The full orthology set induced by *G* is represented by the relation graph *R*. The graph $R'$ is an example of a not satisfiable graph, as $\{c_1, b_1, d_1, a_2\}$ induces a $P_4$, while $R''$ is an example of a satisfiable (it has no induced $P_4$), but not *S*-consistent graph (explanation is given in the text)

We adopt the graph representation considered in [14] for full relation sets. A *relation graphR* on a gene family $\Gamma$ is a graph with vertex set $V(R) = \Gamma$, in which we interpret each edge *uv* of the edge set *E*(*R*) of *R* as an orthology relation between *u* and *v*, and each missing edge (non-edge) $uv \notin E(R)$ as a paralogy relation.^1^ Notice that if $s(u) = s(v)$, then $uv \notin E(R)$. The relation graph of a *DS*-tree *G*, denoted by *R*(*G*), is the graph with vertex set ${\mathcal L}(G)$ and edge set ${\mathcal O} (G)$ (for example, see the relation graph *R* in Fig. 2).

A *DS*-tree for a gene family $\Gamma$ leads to a relation graph, but the converse is not always true. A relation graph *R* is *satisfiable* if there exists a *DS*-tree *G* such that $R(G) = R$. The problem of relation graph satisfiability has been addressed in [10]. The following theorem is a reformulation of one of the main results of this paper.

#### **Theorem 1**

([10])* A relation graph R is satisfiable if and only if Ris*$P_4$*-free, meaning that no four vertices of Rinduce a path of length three.*

For example, in Fig. 2, the relation graphs *R* and $R''$ are satisfiable, while the graph $R'$ is not. As a *DS*-tree does not necessarily represent a true history for $\Gamma$ (see previous section and Definition 1), satisfiability of a relation graph does not ensure a possible translation in terms of a history for $\Gamma$. For this to hold, *R* should be *consistent* with the species tree, according to the following definition.

#### **Definition 2**

Given a species tree *S*, a relation graph *R* for $\Gamma$ is *S*-consistent if and only if *R* is satisfiable by a *DS*-tree *G* which is itself *S*-consistent.

For example the graph *R* in Fig. 2 is *S*-consistent. Notice that *S*-consistency implies satisfiability. Results from [14] complete the characterization of *S*-consistent graphs through Theorem 2. A triplet is a binary tree with leafset *L* of size three. For $L = \{x, y, z\}$, we denote by *xy*|*z* the unique triplet *T* on *L* for which $lca_T(x, y) \ne r(T)$ holds. Now $P_3(R)$ is the subset of triplets of species induced by paths having exactly three vertices in $R = (V, E)$:

$$P_3(R) = \{s(x)s(y)|s(z): \; zx, zy \in E \; \text{ and }\; xy \notin E \; \text{ and }\; s(x) \ne s(y) \}$$

We present in Theorem 2 a necessary and sufficient condition for *S*-consistency of a relation graph in terms of $P_3(R)$. First, we introduce in Lemma 2 an intermediate property, that is useful for proving Theorem 2.

#### **Lemma 2**

*Let G be a DS-tree and S be a species tree. Then for any internal node v of G, there exist leaves x, y of *$G_v$* such that both the following hold: (1) *$lca_S(s(x), s(y)) = s_G(v)$* and (2)*$lca_G(x, y) = v$.

#### Proof

We first show that (1) must hold for some $x, y \in {\mathcal L}(G_v)$. If $s_G(v)$ has two children $s_1$ and $s_2$ for which there exist leaves *x* and *y* of $G_v$ such that $s_1$ is an ancestor of *s*(*x*) and $s_2$ an ancestor of *s*(*y*), then (1) holds. Thus if we suppose that (1) does not hold, then $s_G(v)$ has a child $s'$ such that all leaves of $G_v$ belong to a species that has $s'$ as an ancestor. This implies that $s'$ is a lower common ancestor than $s_G(v)$ for the species present in $G_v$, contradicting the definition of $s_G$.

Now, take *x* and *y* satisfying (1). Suppose that (2) does not hold for *x* and *y*, i.e. $lca_S(s(x), s(y)) = s_G(v)$, but that $lca_G(x, y) \ne v$. Take $z \in {\mathcal L}(G_v)$ such that *z* is separated from $lca_G(x, y)$ by *v* (i.e. $lca_G(z, lca_G(x, y)) = v$). We have $lca_G(x, z) = lca_G(y, z) = v$. If $lca_S(s(x), s(z)) = s_G(v)$, then we are done as *x* and *z* satisfy both (1) and (2). Otherwise, $lca_S(s(x), s(z))$ is on the $s(x) - s_G(v)$ path, implying that $lca_S(s(y), s(z)) = s_G(v)$ since $s_G(v)$ separates *s*(*x*) from *s*(*y*). In this last case, *y* and *z* are the leaves of interest, ending the proof. $\square$

#### **Theorem 2**

*Let*$R = (V,E)$* be a satisfiable relation graph and let S be a species tree. Then R is S-consistent if and only if S displays all the triplets of *$P_3(R)$.

#### Proof

$\Rightarrow$ : let *G* be an *S*-consistent gene tree satisfying *R*, and let $x, y, z \in V(R)$ such that $zx, zy \in E(R)$ but $xy \notin E(R)$ and $s(x) \ne s(y)$. Then we must have $zx, zy \in {\mathcal O} (G)$ and $xy \in {\mathcal P} (G)$. We claim that *S* must display the *s*(*x*)*s*(*y*)|*s*(*z*) triplet. Let $\alpha = lca_G(x, y), \beta = lca_G(x, z)$ and $\gamma = lca_G(y, z)$. Since $ev_G(\alpha ) \ne ev_G(\beta ) = ev_G(\gamma )$, *xy*|*z* must be a triplet of *G*. Moreover, since $ev_G(\gamma ) = ev_G(\beta ) = Spec$, $lca_S(s(x), s(y))$ and *s*(*z*) must be separated in *S*, implying that *s*(*x*)*s*(*y*)|*s*(*z*) is a triplet of *S*.

$\Leftarrow$ : by assumption, *R* is satisfiable by some *DS*-tree $G'$. We first obtain from $G'$ a least-resolved *DS*-tree *G* satisfying *R*, in terms of speciation. That is, if $G'$ has any speciation node *v* that has a speciation child *w*, we obtain $G''$ by contracting *v* and *w* (delete *w* and give its children to *v*). Note that we have ${\mathcal O} (G') = {\mathcal O} (G'')$ and so $G''$ still satisfies *R*. We obtain the *DS*-tree *G* by repeating this operation until we cannot find such a *v* and *w*. We claim that if *S* displays the triplets of $P_3(R)$, then *G* is *S*-consistent.

Let *v* be a speciation node of *G*, and let $v_1, v_2$ be any two distinct children of *v*. By the construction of *G*, $ev_G(v_1) = ev_G(v_2) = Dup$. By Lemma 2, $G_{v_1}$ has two leaves $x_1, x_2$ such that $lca_S(s(x_1), s(x_2)) = s_G(v_1)$ and $lca_G(x_1, x_2) = v_1$. Similarly, $G_{v_2}$ has two leaves $y_1, y_2$ with $lca_S(s(y_1), s(y_2)) = s_G(v_2)$ and $lca_G(y_1, y_2) = v_2$. Since *v* is a speciation while $v_1, v_2$ are duplications, we have $x_1x_2, y_1y_2 \notin E(R)$ while $x_1y_1, x_1y_2, x_2y_1, x_2y_2 \in E(R)$. Thus, $x_1y_1x_2$ and $x_1y_2x_2$ are induced paths of length two in *R*, which implies that *S* displays the $s(x_1)s(x_2)|s(y_1)$ and $s(x_1)s(x_2)|s(y_2)$ triplets. Analogously, *S* displays the $s(y_1)s(y_2)|s(x_1)$ and $s(y_1)s(y_2)|s(x_2)$ triplets. This is only possible if $lca_S(s(x_1), s(x_2)) = s_G(v_1)$ and $lca_S(s(y_1), s(y_2)) = s_G(v_2)$ are separated in *S*. We deduce that all child pairs of *v* are from separated species, and hence that *G* is *S*-consistent. $\square$

As an example, the graph $R''$ in Fig. 2 is satisfiable but not *S*-consistent as the path of length 2 containing $\{a_1, b_1, c_1\}$ induces the triplet *ac*|*b*, while the triplet displayed by *S* is *ab*|*c*.

We end this section with additional notations that will be of use later. A *subgraph*$H'$ of *H* is a graph with $V(H') \subseteq V(H)$ and $E(H') \subseteq E(H)$. For a graph *H* and some $V' \subseteq V(H)$, the *subgraph ofHinduced by*$V'$, denoted $H[V']$, is the subgraph of *H* with vertex-set $V'$ having the maximum number of edges. We say that $H'$ is an *induced subgraph of H* if there is a subset $V' \subseteq V(H)$ such that $H' = H[V']$. If *I* is another graph, we say *H* is *I*-free if there is no $V' \subseteq V(H)$ such that $H[V']$ is isomorphic to *I*. Finally, for some edge set $E' \subseteq E(H)$, $H - E'$ is the subgraph $H'$ with $V(H') = V(H)$ and $E(H') = E(H) \setminus E'$.

## Relation correction problems

We raise the issue of leaving out a minimum of information from a relation graph *R* in order to reach satisfiability and *S*-consistency. Two optimality criteria are considered: (1) the minimum number of edges that need to be removed; (2) the maximum number of genes that can be kept.

### The minimum edge-removal consistency problem

Based on the same construction used in paper [34], we show that adding the information on the species tree *S* does not make the problem of removing the minimum number of edges leading to a $P_4$-free graph simpler. Although a similar reduction is likely to hold in the general case of edge-modification (removal or insertion) [31], here we focus on edge removal, as this formulation is needed in subsequent developments. We show the NP-Completeness of this problem, even when every gene from the family $\Gamma$ comes from a distinct species.

**Minimum edge-removal consistency problem:**

**Input:** A relation graph *R* for a gene family $\Gamma$, a species tree *S* and an integer *k*;

**Output:** “Yes” if and only if there exists an *S*-consistent subgraph $R'$ of *R* with $V(R') = V(R)$ such that $|E(R) \setminus E(R')| \le k$.

#### **Theorem 3**

*The *Minimum Edge-Removal Consistency Problem* is NP-Complete, even if for any distinct*$g_1, g_2 \in \Gamma$, $s(g_1) \ne s(g_2)$.

#### *Proof*

Given $R'$ as a certificate, Theorem 2 easily translates into a polynomial-time algorithm to verify that $R'$ is *S*-consistent. It is also clear that verifying if $|E(R) \setminus E(R')| \le k$ can be done quickly. The problem is therefore in NP. As for NP-Hardness, the reduction is from the exact 3-cover problem, a classic NP-Hard problem [35]: given a set $W = \{w_1, \ldots , w_{3t} \}$ and a collection $Z = \{Z_1, \ldots , Z_r\}$ of 3-elements of *W*, does there exists $Z' \subseteq Z$ such that $|Z'| = t$ and $Z'$ is a partition of *W* ? We assume that $r \ge t$.

**Fig. 3:**
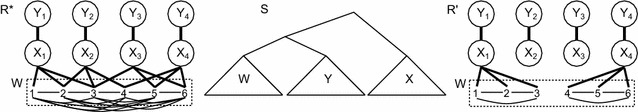
*S* represents the species tree and $R^*$ the relation graph constructed from the sets *W*, *Z*, *X* and *Y*. The illustration is given for $W =\{1,2,3,4,5,6\}$ and $Z = \{\{1,2,3\}, \{2,3,4\}, \{3,5,6\}, \{4,5,6\}\}$. $Z' = \{\{1,2,3\}, \{4,5,6\}\}$ is a subset of *Z* which is a partition of *W*. $R'$ is the “corrected” relation graph corresponding to $Z'$

Given arbitrary *W* and *Z*, we construct *R* and *S* by first defining the species set $\Sigma$. Let $\alpha = {{3t} \atopwithdelims ()2}$ and let $X = \{X_1, \ldots , X_r\}$ and $Y = \{Y_1, \ldots , Y_r\}$ be two collections of all disjoint sets of species (i.e. for any distinct set $A, B \in X \cup Y$, $A \cap B = \emptyset$), with $|X_i| = \alpha$ and $|Y_i| = r^2\alpha$, for all $1 \le i \le r$. Let $X_{\Sigma } = \bigcup _{1 \le i \le r} X_i$ and $Y_{\Sigma } = \bigcup _{1 \le i \le r} Y_i$ be the species in *X* and *Y*. Then the species set is $\Sigma = W \cup X_{\Sigma } \cup Y_{\Sigma }$. Let $S_{W}, S_X$ and $S_Y$ be three trees such that ${\mathcal L}(S_{W}) = W, {\mathcal L}(S_X) = X_{\Sigma }$ and ${\mathcal L}(S_Y) = Y_{\Sigma }$. Then *S* is obtained by first connecting $r(S_Y)$ with $r(S_{W})$ to obtain a new tree $S_{W Y}$, then connecting $r(S_{W Y})$ with $r(S_X)$ (see Fig. 3). Therefore *S* has exactly $|\Sigma | = 3t + r(\alpha + r^2\alpha )$ leaves. The gene family $\Gamma$ is then constructed so that it contains exactly one gene per species, as mentioned in the Theorem statement. In other words the mapping $s : \Gamma \rightarrow \Sigma$ is a bijection. Thus for simplicity, we make no distinction between a gene *g* and its species *s*(*g*). We then define *R* with $V(R) = \Sigma$ such that each of the sets $W, X_1, \ldots , X_r, Y_1, \ldots , Y_r$ forms an individual clique. Finally we add two edge-sets $E_1$ and $E_2$ to *R*, where $E_1 = \{g_1g_2 : g_1 \in X_i, g_2 \in Z_i, \; \text{ for } \text{ a } \text{ given } \;1 \le i \le r\}$ and $E_2 = \{g_1g_2 : g_1 \in X_i, g_2 \in Y_i, \; \text{ for } \text{ a } \text{ given } \;1 \le i \le r\}$. Then *R* has $2r + 1$ cliques, namely $W, X_1, \ldots , X_r, Y_1, \ldots , Y_r$. Also, for $1 \le i \le r$, all edges between $X_i$ and $Y_i$ are present, as well as all edges between $X_i$ and $Z_i$. Figure 3 gives an example with $t=2$ and $W = \{1,2,3,4,5,6\}$.

Notice that the construction of *R* described above can clearly be done in polynomial time. We now show that *W* and *Z* admit an exact 3-cover if and only if *R* admits an *S*-consistent *DS*-tree after the deletion of at most $3\alpha (r - t) + (\alpha - 3t)$ edges.

($\Rightarrow$) : let $Z' \subseteq Z$ be a partition of *W*, $|Z'| = t$. Let $R'$ be the subgraph of *R* in which all edges between $Z_i$ and $X_i$ are removed if and only if $Z_i \notin Z'$ (which removes $3\alpha (r - t)$ edges), and the only edges not removed from the *W*-clique are those belonging to a $Z_i$ triangle with $Z_i \in Z'$ (which removes $\alpha - 3t$ edges). An example of $R'$ is given in Fig. 3. Thus there are exactly $3\alpha (r - t) + (\alpha - 3t)$ edges of *R* missing from $R'$, as desired. Clearly, $R'$ is $P_4$-free and thus satisfiable. To see that $R'$ is *S*-consistent, we use Theorem 2. Notice that any path of length 3 in $R'$ has the form $wx_iy_i$ with $w \in W, x_i \in X_i$ and $y_i \in Y_i$ for some *i*, inducing the $wy_i|x_i$ speciation triplet, which is in agreement with *S*. Therefore there exists an *S*-consistent gene tree $G'$ satisfying $R'$.

($\Leftarrow$) : let $R'$ be an *S*-consistent relation graph obtained by deleting at most $3\alpha (r - t) + (\alpha - 3t)$ edges from *R*. Then, $R'$ must be $P_4$-free. We show that $R'[W]$ is partitioned into triangles which form a solution to the 3-cover instance. Let $w \in W$. We claim that in $R'$, there is **exactly** one $X_i \in X$ such that *w* has neighbors in $X_i$. Suppose first there are $x_1 \in X_i$ and $x_2 \in X_j$, $i \ne j$, such that both $x_1$ and $x_2$ are neighbors of *w* in $R'$. Then there is some $y \in Y_i$ such that $yx_1wx_2$ induce a $P_4$, unless all edges between $x_1$ and $Y_i$ were deleted. But we reach a contradiction since there are $r^2\alpha > 3\alpha (r - t) + (\alpha - 3t)$ such edges. Therefore *w* has neighbors in at most one $X_i \in X$. Using that fact, we can see that *w* must have at least one neighbor in *X*, since otherwise at most $(3t - 1)\alpha$ edges between *X* and *W* would remain, implying the deletion of $3 \alpha r - (3t - 1) \alpha = 3\alpha (r - t) + \alpha$ edges, more than permitted. This proves our claim.

Thus at best, each $w \in W$ has $\alpha$ neighbors in *X*, implying that at least $3 \alpha r - 3t \alpha = 3\alpha (r - t)$ deleted edges are between *X* and *W*. This leaves a maximum of $\alpha - 3t$ other edges that can be deleted.

Now, let *C* be a connected component of $R'[W]$. We claim that all vertices of *C* must have their *X* neighbors in the same $X_i \in X$. For suppose otherwise that there are two vertices $c_1, c_2$ of *C* such that $c_1$ has a neighbor $x_1 \in X_i$ and $c_2$ a neighbor $x_2 \in X_j$ with $i \ne j$. It is easy to see that such $c_1$ and $c_2$ can be chosen to be neighbors. Then $x_1, c_1, c_2, x_2$ induce a $P_4$, a contradiction. Thus all vertices of *C* have their *X* neighbors in a common $X_i \in X$. Since each vertex of $X_i$ has three neighbors in *W*, this implies that *C* has at most three vertices. Suppose that *C* that has two vertices or less. Then since all vertices of $R'[W]$ have at most two neighbors, it can have at most $\frac{1}{2}(2(3t - 2) + 2) = 3t - 1$ edges (obtained by counting the sum of degrees). This, however, implies that at least $\alpha - (3t - 1)$ additional edges were deleted, more than the $\alpha - 3t$ available.

We conclude that $R'[W]$ is partitioned into *t* connected components, each having three vertices. Moreover, each vertex in a given component *C* has neighbors in the same $X_i \in X$, implying that $Z_i$ contains the members of *C*. Finally since the components are all associated with a disctinct $Z_i$, $R'[W]$ effectively defines a solution to the exact cover instance. $\square$

### The Maximum Node Consistency problem

We introduce the Maximum Node Consistency Problem (in its decision version) and we consider the approximation complexity of the corresponding optimization version.

**Maximum Node Consistency problem:**

**Input:** A relation graph *R* for a gene family $\Gamma$, a species tree *S* and an integer *k*;

**Output:** “Yes” if and only if there exists an *S*-consistent induced subgraph $R'$ of *R* with $|V(R')| \ge k$.

We show that Maximum Node Consistency is hard to approximate within a factor $d n^{\frac{1}{2}(1-\varepsilon )}$ for any $0 < \epsilon < 1$ and any constant $d>0$, by giving a gap-preserving reduction from Maximum Independet Set (*n* is the number of nodes of *R*). We refer the reader to [36] for a definition of gap-preserving reduction. Consider an instance $H=(V_H,E_H)$ of Maximum Independet Set, with $|V_H|=m$. We construct an instance of Maximum Node Consistency as follows.

First, we define the set of genes $\Gamma$, i.e. the nodes of the relation graph *R*. Denote $V_H = \{v_1, \ldots , v_m\}$ and for each $v_i \in V_H$, we define a set $I(v_i)$ of *m* genes: $I(v_i)= \{ r_{i,j}: 1 \le j \le m \}$. The gene set $\Gamma$ is $\bigcup _{v_i \in V_H} I(v_i)$.

Now, we define the species tree *S*. First consider *S* as any binary tree over *m* leaves ${\ell }_1, \ldots , {\ell }_m$, and replace each leaf ${\ell }_i$ by any binary subtree $T_i$ having *m* leaves (thus *S* has $m^2$ leaves). Each gene in $I(v_i)$ is mapped to a leaf of $T_i$ in a bijective manner, and so each species has exactly one gene in *R*. We make no distinction between $g \in \Gamma$ and *s*(*g*).

Now, define the relation graph $R=(V_R,E_R)$. Set $V_R = \Gamma$, and we get that $n = |V_R| = m^2$. For each $v_i \in V$, $I(v_i)$ forms a clique in *R*. Moreover, for each $\{v_i,v_j\} \in E_H$, define an edge $\{ r_{i,t}, r_{j,t} \} \in E_R$, for each *t* with $1 \le t \le m$.

Let $R'$ be a solution of Maximum Node Consistency over instance (*R*, *S*). Denote by $R'(v_i)$ the subset of nodes $V(R') \cap I(v_i)$, that is those nodes of $I(v_i)$ that have not been removed. We pay a particular attention to those $R'(v_i)$ that contain more than one node.

#### **Lemma 3**

*Let *$R'(v_i), R'(v_j)$* be two subsets of nodes of a solution *$R'$*of *Maximum Node Consistency *over instance (R, S) such that *$|R'(v_i)| \ge 2$* and *$|R'(v_j)| \ge 2$*. Then there is no edge with one endpoint in*$R'(v_i)$* and the other in *$R'(v_j)$.

#### Proof

Assume on the contrary that there is some *q* such that $r_{i,q} \in R'(v_i)$ and $r_{j,q} \in R'(v_j)$ share an edge. Consider a node $r_{i,z}$ of $R'(v_i) \setminus \{ r_{i,q} \}$, which must exist since $|R'(v_i)| \ge 2$. The $P_3$ induced by $r_{i,z}, r_{i, q}$ and $r_{j, q}$ implies the triplet $(r_{i,z},r_{j,q}|r_{i,q})$, while *S* contains the triplet $(r_{i,z},r_{i,q}|r_{j,q})$. $\square$

Now, we are ready to prove the main result of this section.

#### **Lemma 4**

*Let a graph H be an instance *Maximum Independet Set* with m nodes, and let (R, S) be the corresponding instance of *Maximum Node Consistency*with *$n = m^2$* nodes. Then*

1. *Given an independent set *$V'$* of H, we can compute in polynomial time a solution of *Maximum Node Consistency* of size at least *$|V'|m$;
2. *Given a solution of *Maximum Node Consistency *on instance (R, S) of size at least k m, we can compute in polynomial time an independent set *$V'$*of H such that*$|V'| \ge k$.

#### *Proof*

1. Consider an independent set $V'$ of *H* and define a solution of Maximum Node Consistency on instance (*R*, *S*) of size at least $|V'|m$ as follows: remove each node of $I(v_i)$ if and only if $v_i \notin V'$. Let $R'$ be the corresponding solution of Maximum Node Consistency. Since $V'$ is an independent set, it follows that $R'$ consists only of cliques $R'(v_i)$, disconnected one from the other. It has $|V'|m$ nodes and as $R'$ is $P_3$-free, it is *S*-consistent.
2. The case $k = 1$ is trivial so we assume $k > 1$. Consider a solution $R'$ of Maximum Node Consistency on instance (*R*, *S*) of size at least *km*, and consider the subsets $R'(v_i)$ in $R'$ such that $|R'(v_i)| > 1$. Notice that we can assume that there exist at least *k* such sets, otherwise $R'$ would contain at most $(k-1)m + m - (k - 1) < km$ nodes.

Given an index *j*, consider the set $R^j= \{ r_{i,j} \in R'(v_i): |R'(v_i)| > 1, 1 \le i \le m \}$, i.e. the nodes with index *j* that belong to some subset $R'(v_i)$ larger than one. By Lemma 3 each set $R^j$ is an independent set. Now, pick the set $R^j$ having maximum cardinality. It follows that $R^j$ contains at least *k* nodes, since otherwise $R'$ would have at most $m(k - 1) + m - k < mk$ nodes. Hence, $V'= \{v_i: r_{i,j} \in R^j \}$ is an independent set of size at least *k*, thus concluding the proof. $\square$

We say a maximization problem cannot be approximated within a factor $\alpha$ if, unless $P = NP$, for any approximation algorithm ${\mathcal A}$ there are infinitely many instances for which ${\mathcal A}$ outputs a solution with value *AP* such that $AP < \frac{1}{\alpha } OPT$, where *OPT* is the optimal value of a solution to the problem (note that equivalently, $\frac{OPT}{AP} > \alpha$). It is well-known that Maximum Independet Set cannot be approximated within a factor $cm^{1 - \varepsilon }$ for any $0 < \varepsilon < 1$ and for any constant $c > 0$ [37].

#### **Theorem 4**

*The optimization version of *Maximum Node Consistency* cannot be approximated within a factor*$d n^{\frac{1}{2}(1-\varepsilon )}$* for any *$0 < \varepsilon < 1$* and for any constant *$d>0,$*where n is the number of nodes of the given relation graph. Moreover, this result holds even on instances in which for any distinct *$g_1, g_2 \in \Gamma$, $s(g_1) \ne s(g_2)$.

#### Proof

Let *H* be a graph with *m* nodes and let (*R*, *S*) be the corresponding instance of Maximum Node Consistency with $n = m^2$ nodes. Denote by $OPT_I$ and $OPT_N$, respectively, the values of an optimal solution for Maximum Independet Set and Maximum Node Consistency. Let ${\mathcal A}_N$ be any approximation algorithm for Maximum Node Consistency, and let ${\mathcal A}_I$ be the approximation algorithm for Maximum Independet Set that on input *H*, runs ${\mathcal A}_N$ on the corresponding instance (*R*, *S*) and returns the independent set resulting from Lemma 4. Let $AP_I(H)$ and $AP_N(R, S)$ denote, respectively, the sizes of the solutions found by ${\mathcal A}_I(H)$ and ${\mathcal A}_N(R,S)$. By Lemma 4 we get that $AP_I(H) \ge \lfloor AP_N(R, S)/m \rfloor \ge AP_N(R, S)/m - 1$ and $OPT_N(R,S) \ge OPT_I(H)m$. Now,

$$\begin{aligned} \frac{OPT_N(R,S)}{AP_N(R,S)} \ge \frac{OPT_I(H)m}{AP_I(H)m + m} =\frac{OPT_I(H)}{AP_I(H)+1} \ge \frac{OPT_I(H)}{2AP_I(H)} \end{aligned}$$

as we may assume that $AP_I(H) \ge 1$. Since Maximum Independet Set cannot be approximated within a factor $c m^{1-\varepsilon }$, for any $0 < \varepsilon < 1$ and any constant $c>0$, then for any $0 < \varepsilon < 1$ and any constant $c>0$ there exist infinitely many instances *H* on which $\frac{OPT_I(H)}{2AP_I(H)}~>~cm^{1-\varepsilon }$. Thus, it follows that

$$\begin{aligned} \frac{OPT_N(R,S)}{AP_N(R,S)} \ge \frac{OPT_I(H)}{2AP_I(H)} > \frac{c}{2} m^{1 - \varepsilon } = d n^{\frac{1}{2}(1 - \varepsilon )} \end{aligned}$$

on infinitely many instances. Finally the fact that the result holds even on instances in which for any distinct $g_1, g_2 \in \Gamma$, $s(g_1) \ne s(g_2)$ follows from the construction of *R*. $\square$

We get the following as an immediate corollary, which will be of use later:

#### **Corollary 1**

*The decision version of *Maximum Node Consistency *is NP-Hard, even on instances in which for any distinct *$$g_1, g_2 \in \Gamma,$$$$s(g_1) \ne s(g_2).$$

## Gene tree correction problems

In this section, we are given a gene family $\Gamma$, a species tree *S*, an *S*-consistent *DS*-tree *G* for $\Gamma$, and a set $C = (O, P)$ of orthology/paralogy constraints (not necessarily full). We focus on the problem of correcting *G* according to *C* in a minimal way. The goal is thus to find a *DS*-tree $G'$ inducing *C* such that the difference between *G* and $G'$ is minimum. We consider two ways of measuring the difference (or symetrically the similarity) between gene trees, one based on conserved orthology/paralogy relations induced by the two trees, and one based on the number of conserved clades between the two trees, which is the Robinson-Foulds in the case that *G*, $G'$ and *S* are all binary trees.

### The Maximum Homology Correction problem

**Maximum Homology Correction problem:**

**Input:** A species tree *S*, an *S*-consistent *DS*-tree *G* for a gene family $\Gamma$, an integer *k*, a set *O* of orthology and a set *P* of paralogy relations;

**Output:** “Yes” if there exists an *S*-consistent *DS*-tree $G'$ for $\Gamma$ with $O \subseteq {\mathcal O} (G')$, $P \subseteq {\mathcal P} (G')$ such that $|{\mathcal O} (G) \cap {\mathcal O} (G')| + |{\mathcal P} (G) \cap {\mathcal P} (G')| \ge k$.

#### **Theorem 5**

*The *Maximum Homology Correction*problem is NP-Complete, even if S, G and *$G'$* are required to be binary.*

#### Proof

The problem is clearly in NP, as verifying *S*-consistency can be done in polynomial time, as well as counting the common orthologs/paralogs relations (the set of relations is quadratic in size). For our reduction, we use the Minimum Edge-Removal Consistency problem for the case of a gene family with at most one gene per genome, which is NP-Hard by Theorem 3. Given a species tree *S*, a relation graph *R* with *V*(*R*) in bijection with ${\mathcal L}(S)$ and an integer *k*, we construct an instance of the Maximum Homology Correction Problem, i.e. a species tree $S'$, a *DS*-tree *G*, an orthologous set *O* and paralogous set *P*.

Let $S' = S$ and construct *G* by mimicking *S* - that is by first copying *S* and its leaf labels, then replacing each leaf ${\ell }$ of *G* by the gene $s^{-1}({\ell })$. Note that if *S* is binary, then so is *G*. All internal nodes of *G* are labeled as speciations, so all genes of $\Gamma$ are pairwise orthologous. Thus *R*(*G*) is a clique. Finally, let $O = \emptyset$ and $P = \{g_1g_2 : g_1g_2 \notin E(R) \}$. Therefore the objective is to break a minimum of orthologies of *G* in order to satisfy *P*.

We show that that there is an *S*-consistent subgraph $R'$ of *R* obtained by removing at most *k* edges if and only if there is an $S'$-consistent *DS*-tree $G'$ satisfying *O* and *P* with at most $|P| + k$ relations that are not induced by *G*.

$\Rightarrow$ : Let $R'$ be a solution to the Minimum Edge-Removal Consistency Problem for *R* and *S*. Then there exists a *S*-consistent *DS*-tree $G'$ satisfying $R'$, which is obtained by deleting at most *k* edges from *R*. By Lemma 1, we may assume that if *S* is binary, then so is $G'$. Now, since $R'$ has at most $|P| + k$ non-edges, $G'$ has at most $k + |P|$ paralogs and is therefore a solution to the constructed instance of the Maximum Homology Correction Problem that breaks at most $k + |P|$ orthologies of *R*(*G*).

$\Leftarrow$ : Let $G'$ be a solution, binary or not, to the constructed Maximum Homology Correction Problem instance and let $R' = R(G')$. Since $G'$ satisfies *P* and breaks at most $|P| + k$ orthologies, $R'$ must have *P* as non-edges, plus at most *k* other non-edges. Thus $R'$ can be obtained by removing at most *k* edges from $R(G) - P = R$, as desired. $\square$

### The maximum clade correction problem

**Maximum clade correction problem:**

**Input:** A gene tree *G*, a species tree *S*, a set *O* of orthology and a set *P* of paralogy relations and an integer *k*;

**Output:** “Yes” if there exists an *S*-consistent *DS*-tree $G'$ satisfying *O* and *P* such that *G* and $G'$ have at least *k* clades in common.

Notice that if *S*, *G* and $G'$ are required to be binary, the effective measure between *G* and $G'$ is the Robinson-Foulds distance. This special case is handled as part of the general proof. But before we need the following lemma, which uses *grafting* operations to add leaves to *G* and satisfy a prescribed relation without breaking other relations.

Given two trees $T_1$ and $T_2$, *connecting*$T_1$ with $T_2$ corresponds to creating a new node *x* and giving it $r(T_1)$ and $r(T_2)$ as its two children. *Grafting* a new leaf *x* to a tree *T* corresponds to adding *x* to ${\mathcal L}(T)$ by either: (1) adding *x* as a new child of some node *u* of *T*; (2) connecting *T* with *x*; (3) subdividing an edge *uv* and adding *x* as a child of the newly created vertex.

#### **Lemma 5**

*Let G be an S-consistent gene tree, for some species tree S. Let x be a gene not in G and y be some gene in G with *$s(x) \ne s(y)$.* Then there exists a gene tree *$G'$*obtained by grafting x to G such that the following conditions are satisfied:*

1. *x and y are orthologs in *$G'$;
2. $${\mathcal O} (G) \subseteq {\mathcal O} (G')$$* and*$${\mathcal P} (G) \subseteq {\mathcal P} (G')$$;
3. $$G'$$*is S-consistent;*

#### Proof

If $s_G(r(G))$ is a strict descendant of $lca_S(s(x), s(y))$, then it is easy to see that connecting *x* to *r*(*G*) under a common parent yields the desired result. So we assume $s_G(r(G))$ is an ancestor of $lca_S(s(x), s(y))$. If there is some node *u* of *G* such that adding *x* as a child of *u* satisfies the three conditions of the Lemma, then we are done. So assume that there is no node *u* to which we can add *x* as a child.

Let *uv* be an edge of *G*, and suppose that we graft  *x* on *uv* to obtain $G'$. Call *p* the parent of *x* on $G'$, and say that *u* is the parent of *p* (i.e. *p* has children *x* and *v*). Note that if $s_G(u) = s_{G'}(u)$, then $s_G(w) = s_{G'}(w)$ for any $w \in V(G) \cap V(G')$, implying that setting $ev_{G'}(z) = ev_{G}(z)$ for all $z \in V(G) \cap V(G') \setminus \{u\}$ preserves *S*-consistency. We will find such a *uv* that guarantees this $s_G(u) = s_{G'}(u)$ property, while ensuring that $lca_{G'}(x, y)$ can be a speciation (i.e. $ev_{G'}(lca_{G'}(x, y)) = Spec$ is *S*-consistent), and that $ev_G(u) = ev_{G'}(u)$ is *S*-consistent. This will prove the Lemma.

Let $s_{xy} = lca_S(s(x), s(y))$, and let *g* be the lowest ancestor of *y* in *G* such that $s_G(g)$ is $s_{xy}$ or an ancestor of $s_{xy}$. Note that the case in which *g* does not exist was handled in the beginning of the proof. Now suppose that $ev_{G}(g) = Dup$. Denote by $g'$ the child of *g* that is also an ancestor of *y*. Note that $s_G(g)$ is an ancestor of $s_{xy}$ and $s_G(g')$ is a strict descendant of $s_{xy}$. We claim that $uv = gg'$. To see this, obtain $G'$ by grafting *x* to $gg'$, *p* being the parent of *x* and *g* the parent of *p*. Then, $s_{G'}(p) = s_{xy}$, and its children species $s_{G'}(g')$ and $s_{G'}(x)$ are separated in *S* by our choice of $g'$. Thus setting $ev_{G'}(p) = ev_{G'}(lca_{G'}(x, y)) = Spec$ preserves *S*-consistency. Also, $s_G(g) = s_{G'}(g)$ since $s_G(g)$ is already an ancestor of $s_{xy} = s_{G'}(p)$. Finally, we are free to set $ev_{G'}(g) = Dup$, satisfying all the required conditions.

So instead suppose that $ev_G(g) = Spec$. Recall that adding *x* as a child of *g* to obtain a new tree $G''$ is not a solution. Since in $G''$, $lca_{G''}(x, y) = g$, we must either have $s_G(g) \ne s_{G''}(g)$, or $ev_{G''}(g) = Spec$ is not *S*-consistent. By the choice of *g*, only the latter is possible, implying that all children of *g* are from separated species in *G*, but not in $G''$. Therefore, there must be a child $g'$ of *g* such that $s_G(g')$ is an ancestor of *s*(*x*). Note that $g'$ must be unique since otherwise, $ev_G(g) = Spec$ would not be possible. We then claim that $uv = gg'$. Indeed, obtain $G'$ by grafting *x* to $gg'$, *p* being the parent of *x*. We have $s_G(p) = s_{G'}(g')$, and we set $ev_{G'}(p) = Dup$. The species of the children of *g* remain unchanged in $G'$, and so $s_G(g) = s_{G'}(g)$ and $ev_{G'}(g) = ev_{G'}(lca_{G'}(x, y)) = Spec$ is *S*-consistent, again satisfying all required conditions. $\square$

#### **Theorem 6**

*The *Maximum Clade Correction Problem* is NP-Complete, even if S, G and *$G'$* are required to be binary.*

#### Proof

**Fig. 4:**
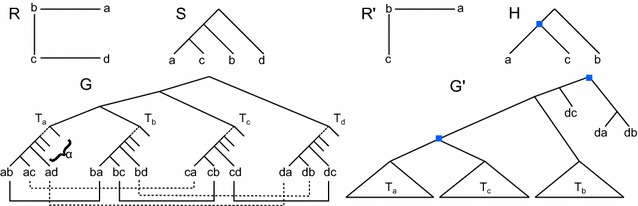
*R* and *S* are the input relation graph and species tree, respectively, for an instance of the Maximum Node ConsistencyProblem (genes of *R* are labeled by their species). The gene tree *G* is constructed from *R* as described in the proof. Leaves of *G* related through orthology in *O* are joined by a black solid edge, while paralogs are joined by a dotted line. The graph $R'$ is a solution to the consistent induced subgraph with $k = 3$, and *H* is the *DS*-tree corresponding to $R'$. The tree $G'$ is a solution to the Maximum Clade Correction Problem constructed from *H*

Verifying *S*-consistency and comparing the set of clades from *G* and $G'$ can clearly be done in polynomial time, thus the problem is in NP. We use the Maximum Node Consistency problem for our reduction, which is NP-Hard by Corollary 1. Let *R*, *S* and *k* be the Maximum Node Consistency instance, letting *R* be the relation graph with $V(R) = \{v_1, \ldots , v_n\}$, *S* the species tree and *k* an integer. Let $\alpha = n(n - 1 - k) + 2k$ (noting that $\alpha > 0$ when $k \le n$). The constructed instance of the Maximum Clade Correction Problem uses the same species tree *S*. Construct *G* as follows: first consider *G* as any binary tree with *n* leaves $l_1, \ldots , l_n$, where each leaf $l_i$ is mapped to vertex $v_i$ of *R*. Then, replace each leaf $l_i$ by a subtree $T_i$ constructed as follows: $T_i$ is a caterpillar tree with $n - 1 + \alpha$ leaves, and each leaf ${\ell }$ of $T_i$ is such that $s({\ell }) = s(v_i)$ (recall that a caterpillar tree is a path to which we add a leaf child to each internal node). Let $L_i$ denote the set of the $n-1$ deepest leaves of $T_i$ (the depth of a leaf ${\ell }$ being the number of nodes on the path between ${\ell }$ and the root). Each leaf of $L_i$ is mapped to a distinct node of $V(R) \setminus \{v_i\}$. Denote by ${\ell }_{i, j}$ the leaf of $T_i$ mapped to $v_j$, and by $N_i$ the subtree of $T_i$ rooted at $lca(L_i)$. Then *G* has exactly $n(n - 1 + \alpha )$ leaves and $n(n - 1 + \alpha ) - 1$ clades (since it is binary). An example is given in Fig. 4. Finally define $O = \{ \{{\ell }_{i,j}, {\ell }_{j,i} \} : v_iv_j \in E(R)\}$ the set of orthology relations to satisfy and $P = \{ \{{\ell }_{i,j}, {\ell }_{j,i} \} : v_iv_j \notin E(R)\}$ the set of paralogy relations to satisfy. Note that each ${\ell }_{i,j}$ is present in exactly one relation.

We show that *R* admits an *S*-consistent induced subgraph with at least *k* nodes if and only if *G*, *O* and *P* admit an *S*-consistent *DS*-tree $G'$ satisfying *O* and *P* such that *G* and $G'$ share at least $k(\alpha + n - 2)$ clades.

($\Rightarrow$) Let $R'$ be a solution to the Maximum Node Consistencyinstance, $|V(R')| \ge k$, and let *H* be a *DS*-tree satisfying $R'$ that is *S*-consistent. By Lemma 1, we may assume that if *S* is binary, then so is *H*. Now, since ${\mathcal L}(H) \subseteq V(R)$, to each leaf $v_i$ of *H* corresponds a subtree $T_i$ in *G*. Then build a *DS*-tree $G^*$ from *H* by replacing each leaf $v_i$ of *H* by $T_i$, and labeling all internal nodes of inserted trees as *Dup* (in Fig. 4, $G^*$ is the subtree of $G'$ rooted at the common ancestor of $T_a, T_b$ and $T_c$). We first argue that $G^*$ is *S*-consistent and satisfies the subsets of *O* and *P* restricted to ${\mathcal L}(G^*)$. In a subsequent step, we will graft the genes missing from $G^*$ using Lemma 5. Notice that $s_H(v_i) = s_{G^*}(r(T_i))$ and that all nodes of *H* that are in $G^*$ have the same LCA-mapping in both trees. It follows that $G^*$ is also *S*-consistent. Also, for all $v_i, v_j \in {\mathcal L}(H)$, $ev_H(lca_H(v_i, v_j)) = ev_{G^*}(lca_{G^*}(r(T_i), r(T_j))$. Thus for any pair of leaves ${\ell }_{i,j}, {\ell }_{j,i}$ in ${\mathcal L}(G^*)$ such that $\{ {\ell }_{i,j}, {\ell }_{j, i} \} \in O$, $lca_{G^*}({\ell }_{i,j}, {\ell }_{j, i})$ is a speciation (by the construction of *O* from *R* and the fact that *H* satisfies $R'$). By the same reasoning, if $\{ {\ell }_{i,j}, {\ell }_{j, i} \} \in P$ then ${\ell }_{i,j}$ and ${\ell }_{j,i}$ are paralogous in $G^*$.

The solution $G'$ is obtained by grafting to $G^*$ every leaf of *G* missing from $G^*$ whilst preserving the $T_i$ clades, maintaining satisfiability of *O* and *P* and *S*-consistency. If such a $G'$ exists, then *G* and $G'$ share at least *k* identical subtrees from $\{T_1, \ldots , T_n\}$, and since each $T_i$ contains $\alpha + n - 2$ clades, it follows that *G* and $G'$ share at least $k(\alpha + n - 2)$ clades as required. Let $L = {\mathcal L}(G) \setminus {\mathcal L}(G^*)$ be the leaves yet missing from $G^*$. Let $L_O = \{{\ell }\in L : \exists {\ell }' \in {\mathcal L}(G^*), {\ell }{\ell }' \in O\}$ (i.e. the leaves of *L* subject to an orthology constraint with some leaf already in $G^*$). The complement $\overline{L_O}$ is the set of leaves of *L* that are either subject to a paralogy constraint with some leaf of $G^*$, or not subject to any constraint with any leaf of $G^*$. Let $R(\overline{L_O})$ be the relation graph with vertex set $\overline{L_O}$ and edge set $\{{\ell }_1{\ell }_2 : {\ell }_1, {\ell }_2 \in \overline{L_O}, {\ell }_1{\ell }_2 \in O\}$, depicting the required orthologies within $\overline{L_O}$. Recall that each leaf of *L* is contained in at most one relation, implying that each node of $R(\overline{L_O})$ has maximum degree 1. Thus $R(\overline{L_O})$ is $P_3$-free and therefore is *S*-consistent. Let $G_{L_O}$ be a *DS*-tree satisfying $R(\overline{L_O})$ that is *S*-consistent, assuming that $G_{L_O}$ is binary if *S* is. We update $G^*$ by joining $r(G^*)$ and $r(G_{L_O})$ under a common parent *x*, and labeling *x* as *Dup*. Notice that this does not modify any orthology or paralogy relation previously in $G^*$ or in $G_{L_O}$, nor does it break *S*-consistency. This also ensures that paralogies ${\ell }_1{\ell }_2 \in P$ with ${\ell }_1 \in \overline{L_O}$ and ${\ell }_2 \in {\mathcal L}(G^*)$ are satisfied.

The final step is to graft the leaves of $L_O$ to $G^*$ in a way satisfying orthology requirements. This is done by successively applying Lemma 5 to each ${\ell }\in L_O$. As shown, each such ${\ell }$ can be grafted into $G^*$ without modifying any orthology or paralogy relation already in $G^*$ whilst satisfying the orthology requirement that ${\ell }$ is subject to. It is straightforward to see that in addition, ${\ell }$ can be grafted without breaking any $T_i$ clade present in $G^*$, since every vertex in $T_i$ is mapped to the same species. The tree $G'$ obtained after all these grafting operations, satisfies every *O* and *P* and has the required common clades with *G*.

($\Leftarrow$) Let $G'$ be a solution, binary or not, to the Maximum Clade Correction Problem instance. Denote by *C* the number of clades shared by both *G* and $G'$, with $C \ge k(\alpha + n - 2)$. Recall that $L_i$ is the set of the $n - 1$ deepest leaves of $T_i$ in *G*, with $N_i$ being the subtree rooted at $lca_G(L_i)$. Denote by $G'_{L_i}$ the subtree of $G'$ rooted at $lca_{G'}(L_i)$. We say that $N_i$ was *preserved* if every leaf of $G'_{L_i}$ belongs to ${\mathcal L}(T_i)$ (in other words, the $N_i$ clade might have been extended, but only to include other leaves from $T_i$). We claim that at least *k* of the $N = \{N_1, \ldots , N_n\}$ subtrees are preserved in $G'$. Assume, on the contrary, that at least $n - k + 1$ subtrees from *N* are not preserved. Take a non-preserved subtree $N_i$. Then some leaf ${\ell }\notin {\mathcal L}(T_i)$ belongs to the $lca_{G'}(L_i)$ clade. This implies that for any ancestor *x* of $r(N_i)$ in *G*, $G'$ cannot contain the *x* clade. By construction of $T_i$, $r(N_i)$ has at least $\alpha$ ancestors in *G*. Therefore, $C \le n(n - 1 + \alpha ) - 1 - \alpha (n - k + 1)$. This leads to $k(\alpha + n - 2) \le C \le n(n - 1 + \alpha ) - 1 - \alpha (n - k + 1)$, and then to $\alpha \le n(n - 1 - k) + 2k - 1$, contradicting our choice of $\alpha$.

Now, let $N^p = \{N_i \in N : N_i$ is preserved in $G'\}$. We have $|N^p| \ge k$. Let $L = \bigcup _{\{i:N_i \in N^p\}} L_i$ and $H = G'|_{L}$. Notice that to each $N_i \in N^p$ corresponds exactly one subtree $N'_i$ in *H* such that ${\mathcal L}(N_i) = {\mathcal L}(N'_i)$ (and all such $N'_i$ subtrees are disjoint). Let $H^*$ be the tree obtained by replacing every subtree $N'_i$ in *H* by $v_i$. Replacing $N'_i$ by $v_i$ changes no LCA-mapping value since all vertices of $N'_i$ map to $s(v_i)$. Thus as $G'$ is *S*-consistent, then so are *H* and $H^*$. Now, we claim that $H^*$ induces the set of relations represented by $R' = R[{\mathcal L}(H^*)]$, which proves the theorem since $|{\mathcal L}(H^*)| = |N^p| \ge k$. By contradiction, suppose that $v_iv_j \in E(R')$ but $lca_{H^*}(v_i, v_j)$ is labeled *Dup*. Then $lca_H({\ell }_{i,j}, {\ell }_{j,i})$ is also labeled *Dup*, and so is $lca_{G'}({\ell }_{i,j}, {\ell }_{j, i})$. But ${\ell }_{i,j} {\ell }_{j,i} \in O$, contradicting our assumption that $G'$ is a solution. The same reasoning applies when $v_iv_j \notin E(R')$, ending the proof. $\square$

## Algorithmic avenues

As the problems considered in this paper are all computationally hard, only non-polynomial exact algorithms or approximation algorithms avenues can realistically be explored. Let us generalize the Minimum Edge-Removal Consistency problem to the minimum *editing* problem (i.e. minimzing edge removals and insertions). It is not hard to imagine a branch-and-bound algorithm that solves the problem. Call an induced subgraph *H* of a relation graph *Rbad* if it is a $P_4$, or there is triplet of $P_3(H)$ not displayed by *S*. Each $P_4$ can be solved by six possible edge editings, and each contradictory triplet of $P_3(H)$ can be solved by three possible editings. Therefore, in a branch-and-bound process, one would verify if a given graph $R'$ contains a bad subgraph and if so, proceed recursively on each graph obtained by an editing that removes it. If no bad subgraph exists, then $R'$ is a possible solution and its number of editings is retained. If, at any point, $R'$ has had more editings than the best solution encountered so far, the algorithm can stop the recursion. Notice however that an edge should not be edited more than once in order to avoid infinite loops. The idea of this branch-and-bound algorithm can also be applied to the Maximum Node Consistency problem. It is known that a $P_4$, if one exists, can be found in linear time [38], and clearly a contradictory triplet, if any, can be found in time $O(n^3)$ (though a more efficient algorithm may exist). A similar approach has been applied in [31] to design an FPT algorithm for the satisfiability problem.

As for approximations, an algorithm proposed in [32] can be directly applied to the Minimum Edge-Removal Consistency problem and guarantees that we do not remove more than $4 \Delta (R)$ times more edges than the optimal solution, where $\Delta (R)$ is the maximum degree of *R*. The idea is simple: as long as *R* has a bad subgraph *H*, remove every edge incident to a vertex of *H* and continue. Even though this is the best known approximation algorithm so far, it has the undesirable effect of isolating many vertices, motivating the exploration of alternative algorithms. One direction would be to consider existing ideas on the problem of satisfiability, i.e. finding the minimum number of editings required to make a graph $P_4$-free, and adapt them to the consistency problem - for instance the Min-Cut algorithm proposed in [39].

As for gene tree correction, we have developed in [14] a polynomial-time algorithm which, given a species tree *S* and partial sets of relations *O* and *P*, verifies if there exists an *S*-consistent gene tree $G'$ satisfying *O* and *P* and if so, constructs one among the set of all possible solutions. In ordre to correct a gene tree *G*, we can envisage an extension of this algorithm allowing to provide *G* as input, and pick, among the solutions of the algorithm the one which is the closest to *G* (either in terms of common homology relations or clades).

## Conclusions

A gene tree induces a set of orthology and paralogy relations between members of a gene family, but the converse is not always true. In this paper we have shown that attempting to modify a set of relations as least as possible in order to ensure consistency with a species tree leads to the formulation of NP-Complete problems. Moreover, even assuming that the given relations are error-free, it remains computationally difficult to correct a gene tree in order to fit the given set of relations. As various model-free methods are available to infer orthology and paralogy, these correction problems are of practical biological interest. A future direction would be to explore the exact branch-and-bound algorithms and heuristics mentioned in the last section, and design fast approximation algorithms for the relation graph and gene tree editing problems.

## Footnotes

1 The term ‘relation graph’ is also used in phylogenetics in the form of a generalization of a median network to a set of partitions. To make it clear, relation graphs in this paper have nothing to do with this notion.
